# A comprehensive analysis of chromosomal polymorphic variants on reproductive outcomes after intracytoplasmic sperm injection treatment

**DOI:** 10.1038/s41598-023-28552-w

**Published:** 2023-01-24

**Authors:** Madara S. B. Ralapanawe, Sugandika L. Gajaweera, Nishendra Karunaratne, Vajira H. W. Dissanayake, Malcolm J. Price, Pedro Melo, Arri Coomarasamy, Ioannis D. Gallos

**Affiliations:** 1 Tommy’s National Centre for Miscarriage Research, Institute of Metabolism and Systems Research, Institute of Translational Medicine (ITM), University of Birmingham, 4th floor, Edgbaston, Birmingham, B15 2TT UK; 2 Fertility Centre, Lanka Hospitals Corporation Plc, 578, Elvitigala Mawatha, Colombo, 00500 Sri Lanka; 3 Department of Anatomy, Genetics and Biomedical Informatics, Faculty of Medicine, University of Colombo, Colombo, 00800 Sri Lanka; 4 Institute of Applied Health Research, University of Birmingham, Birmingham, B15 2TT UK; 5 NIHR Birmingham Biomedical Research Centre, University Hospitals Birmingham NHS Foundation Trust, University of Birmingham, Birmingham, B15 2TH UK

**Keywords:** Infertility, Genetics

## Abstract

Recent studies suggest that chromosomal polymorphic variations are associated with infertility. A systematic review of chromosomal polymorphisms in assisted reproduction found an association with higher rates of miscarriage. Aim of this study is to analyse the influence of specific types or number of chromosomal polymorphic variations on reproductive outcomes of couples undergoing ICSI treatment. We analysed data from 929 fresh and frozen embryo transfer cycles of 692 women who underwent karyotyping analysis using Giemsa-Trypsin-Leishman (GTL) banding prior to the ICSI procedure at the Fertility Centre of Lanka Hospitals Corporation Plc, Sri Lanka, from January 2016 to December 2018. The outcomes of interest were the pregnancy, miscarriage and live birth rate per cycle. There was no evidence of a difference in the reproductive outcomes between carriers or non-carriers of any type or number of chromosomal polymorphic variation. Our data, in contrast to previous studies, does not support a deleterious effect for the type or number of chromosomal polymorphic variations on reproductive outcomes. However, additional prospective, adequately powered studies, conducted in multiethnic populations, are required to further investigate whether the detection of chromosomal polymorphic variants prior to assisted conception may in fact be a futile diagnostic tool.

## Introduction

Infertility is considered a critical component of reproductive health and a global public health priority^1^. In-vitro fertilization (IVF) and intracytoplasmic sperm injection (ICSI) are offered as treatment solutions in couples with fertility issues^2^. More than two million IVF and ICSI treatment cycles are carried out worldwide every year^3^. Despite several improvements in these techniques, the live birth rate for each cycle remains low at about 32%^4^.

Chromosomal polymorphic variations occur in 2–5% of the general population and are considered variations of normal^5^. The incidence of polymorphic variations in the infertile population is higher (approximately 10–15%) comparing to the general population, suggesting an association with infertility^5–7^. Chromosomal polymorphic variations are variants in the heterochromatic regions of the chromosome^8^. Heterochromatic regions are the non-coding regions of tandem repeats of DNA and variations in these regions do not result in different phenotypes^9,10^.

The genes necessary for fertility and viability reside in heterochromatin^11^. Heterochromatin contains in the long arm of the non-acrocentric chromosomes, and in the short arm and satellites of the acrocentric chromosomes^12^. The evidence suggest that heterochromatin is not inert and it is essential for cell and organisms’ viability. Heterochromatin plays a role in spindle attachment, movements of chromosome, meiotic paring and cohesion of sister chromatid^13^. The functions of heterochromatin in polymorphic regions may suppress or silence gene expression, which could affect gametogenesis. This impact of polymorphic variations in chromosomes can play an important role in both male and female infertility^5,14^.

These chromosomal polymorphic variations are divided into non-acrocentric, in which includes metacentric and sub-metacentric chromosomes, and acrocentric chromosomes. In the metacentric chromosomes, the centromere lies in the middle of the chromosome. Meanwhile, in the sub-metacentric chromosomes, the centrosome is deviated towards one end of the chromosome dividing the two arms into unequal lengths^15^. According to the international system for human cytogenetics nomenclature (ISCN) standing committee recommendations, autosomes 1 to 3 are the large metacentric chromosomes, 4 and 5 are the large sub-metacentric chromosomes, 6–12 are the medium sizes metacentric and sub-metacentric chromosomes, and 16–20 are the relatively short metacentric and sub-metacentric chromosomes. In non-acrocentric chromosomes, polymorphic variations (heterochromatic segments) are visible on the long arm of the chromosome 1, 9 and 16^5,16^. Pericentric inversions on chromosome 9 [inv (9)] are also considered to be non-acrocentric polymorphic variations (9). In contrast, in the acrocentric chromosome’s centromere lies near the end of the chromosome that one arm is short and other arm is long^15^. According to the ISCN standing committee recommendations, medium sized acrocentric chromosomes with satellites are in chromosome 13, 14 and 15, short acrocentric chromosomes with satellites are in chromosome 21, 22 and Y chromosome without a satellite though heterochromatic segment in the long arm. Satellite stalks and satellites are the acrocentric polymorphic variations frequently occur on the short arms of the chromosome 13, 14, 15, 21 and 22^5,16^.

There is evidence suggesting that specific types of chromosomal polymorphic variations or the presence of multiple chromosomal polymorphic variations influence reproductive outcomes of couples undergoing assisted reproductive technology (ART) treatments^5,17^. A systematic review found that in women with any type or number of chromosomal polymorphic variations undergoing ART treatment, the risk of miscarriage was higher (relative risk [RR] 1.54, 95% confidence interval [CI] 1.19–1.98) than in women with no polymorphic variations undergoing ART treatment^18^. However, studies suggest that specific types of chromosomal polymorphic variations such as non-acrocentric polymorphic variations could adversely affect reproductive outcomes more than other chromosomal polymorphic variations^5,14,19^. The literature is not consistent, and another study suggests that perhaps inversions [Inv (9)] or acrocentric polymorphic variations might lead to lower cleavage rate and increased miscarriage risk^17^.

The current study aims to explore the effects of specific types or the presence of multiple chromosomal polymorphic variations in female partners, male partners and couples on the reproductive outcomes of patients undergoing ICSI treatment.

## Materials and methods

### Study design

This was an analysis recruiting couples where the female partner was aged 19–43 years. Female and male partners underwent karyotyping analysis and a cycle of ICSI treatment prior to embryo transfer at the Fertility Centre of Lanka Hospitals Corporation Plc, Sri Lanka, from January 2016 to December 2018. We followed up pregnancy outcomes until November 2019.

### Exclusion criteria

We excluded participants with incomplete data. We also excluded couples who had an ectopic pregnancy, chromosomal aberrations or non-attendance for of karyotyping testing, substandard follicle development, atypical cleavage of embryos or poor blastocyst formation, donor gametes, elective freeze-all cycles, or non-documented pregnancy outcomes.

### Karyotype analysis

All participants’ peripheral blood leukocytes were karyotyped according to our standard laboratory protocol using Giemsa-Trypsin-Leishman (GTL) banding. Out of the twenty metaphases counted at the banding resolution of 550x, four to five karyotypes were analysed. Two technicians reviewed the karyotyping results independently. The karyotyping group assignment was based on ISCN^16^.

### Follicular stimulation protocols

The female participants underwent controlled ovarian stimulation with either long agonist or short antagonist protocols. The long protocol stimulation was carried out with recombinant FSH 150–450 IU (Gonal F, Merck Serono, Modugno [BA], Italy) along with GnRH agonist downregulation at a dose of 0.1 mg once daily (Decapeptyl, Ferring GmbH, Wittland, Germany). The short protocol stimulated women with similar doses of Gonal F and GnRH antagonist 0.25 mg once daily (Cetrotide, Baxter Oncology GmbH, Halle, Germany) from the eighth day of the stimulation cycle. Ovulation was triggered with hCG 250mcg (Ovidrel, Merck, Serono S.p.A., Modugno (BA), Italy), after the evaluation of the estradiol level on the tenth day of the stimulation protocol (1,000–5,000 pg/ml).

### ICSI, embryo culture and embryo transfer

Oocyte recovery was performed 35 h after the hCG injection followed by the ICSI procedure. All embryos were cultured (Vitrolife Sweden AB, V.Frolunda, Sweden) for up to three days, and at least six and/or more than six cells were selected to transfer on day 3. Routinely two embryos were transferred in the fresh cycle and the remaining embryos were vitrified. A freeze-all policy was undertaken for all who did not have a fresh transfer. Subsequent FET cycles involved warming and transfer at cleavage stage (6–8 cells) or further embryo culture until formation of a blastocyst.

### Pregnancy outcomes and follow-up

The primary outcomes were the pregnancy rate, miscarriage rate and live birth rate per embryo transfer. The pregnancy rate refers to gestational age of 4–6 weeks. The miscarriage rate refers to loss of pregnancy less than 12 weeks of gestational age and live birth rate refers to delivery over 32 weeks of pregnancy. Pregnancy was confirmed by serum β hCG analysis in two weeks from the embryo transfer (serum β HCG > 10mIU/ml). We compared outcomes according to the existence of different chromosomal polymorphic variations including non-acrocentric and acrocentric, a combination of polymorphic variations, as well as the number of polymorphic variations. There were no missing data for clinical characteristics including age, body mass index (BMI), follicular stimulating hormone (FSH) and luteinising hormone (LH) serum levels.

### Statistical analysis

Baseline characteristics and outcome data are described using means with standard deviations for symmetrically distributed continuous data, median with interquartile range for skewed data, and proportions for binary data. We had no missing confounding variable or outcome data. Logistic regression models were fitted to estimate crude and adjusted odds ratios to examine the association of specific types of chromosomal polymorphisms and reproductive outcomes adjusting for confounding variables including age, serum FSH level, serum LH level, BMI and type of treatment (fresh vs frozen). The reference category was no chromosomal polymorphic variations in either partner. All statistical analyses were performed using Stata Statistical Software (Release 16, TX, USA).

### Ethical consideration

Ethical Approval was obtained from the Ethics Committee of Lanka Hospitals Corporation Plc. The procedures used in this study adhere to the tenets of the Declaration of Helsinki. The data of the study collected retrospectively, hence does not require patient consent. Informed consent was obtained from all patients as a routine procedure prior to ICSI treatment at the Fertility Centre of Lanka Hospitals Corporation Plc.

## Results

### Data selection

**Figure 1:**
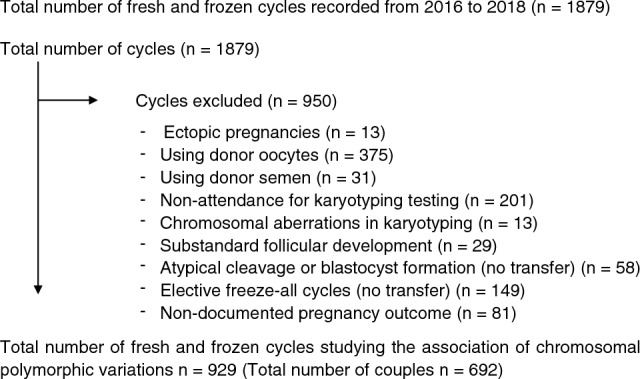
Flow chart of data selection process.

During the study period of January 2016 to December 2018, there were 1,879 fresh and frozen embryo transfer cycles performed at the Fertility Centre. Overall, 950 fresh and FET cycles were excluded from the analysis due to ectopic pregnancies, chromosomal aberrations in karyotyping, absence of karyotyping reports, use of donor gametes, sub-standard follicle development, atypical cleavage of embryos and blastocysts, embryo cryopreservation without transfer and absence of recorded pregnancy outcomes (Fig. 1). There were 149 patients who did not proceed with fresh embryo transfer due to hyperstimulation or any other factors, but went on to have subsequent FET cycles whose outcomes were included in the study. A total of 929 treatment cycles (Fresh 540 and FET 389) from 692 couples were included in the study.

### Baseline characteristics

Table 1 shows the baseline participant characteristics and the treatment types. The mean age, BMI, serum FSH and LH levels were similar in the carriers and non-carriers of non-acrocentric and acrocentric chromosomal polymorphic variations. The proportions of women undergoing long agonist and short antagonist protocols were similar between couples with chromosomal polymorphic variations compared to couples without chromosomal polymorphic variations. The proportions of FET cycles using cleavage stage and blastocyst embryos were also similar between these two groups. Other parameters such as the mean number of oocytes retrieved, mature oocytes, fertilised oocytes and cleavage embryos (day 3) did not differ between the study groups.

**Table 1:** Baseline characteristics and treatment types of the study population.

Characteristics	Couples with chromosomal polymorphic variations n (%) or mean (SD) (n = 490)	Couples without chromosomal polymorphic variations n (%) or mean (SD) (n = 439)
Age	33.7 ± 4.0	34.1 ± 4.2
BMI	24.0 ± 3.4	24.0 ± 4.1
FSH	6.6 ± 1.7	6.6 ± 1.7
LH	5.8 ± 2.8	5.7 ± 2.6
Treatment type		
ICSI cycles		
Long agonist	216 (44.1)	186 (42.4)
Short antagonist	76 (15.5)	62 (14.1)
FET cycles		
Cleavage stage transfers (Day 3)	101 (20.6)	115 (26.2)
Blastocyst stage transfers (Day 5)	97 (19.8)	76 (17.3)
Oocytes retrieved	15.8 ± 8.8	15.2 ± 7.5
Mature oocytes	15.4 ± 8.7	14.5 ± 7.5
Fertilised oocytes	11.7 ± 8.1	10.6 ± 6.3
Cleavage embryos (Day 3)	7.8 ± 5.4	7.0 ± 4.7

*BMI* Body mass index, *FSH* Follicle stimulation hormone, *LH* Luteinising hormone. *ICSI* Intra Cytoplasmic Sperm Injection, *FET* Frozen Embryo Transfer.

### Types of chromosomal polymorphic variations

The distribution of the chromosomal polymorphic variations in females, males and couples are shown in Supplementary Table 1. The prevalence of non-acrocentric and acrocentric chromosomal polymorphic variations from the 929 cycles analysed are shown in Table 2.

**Table 2:** Prevalence of non-acrocentric and acrocentric chromosomal polymorphic variations.

Categories	n (%)
Non-acrocentric female only	24 (2.6)
Acrocentric female only	121 (13.0)
Both non-acrocentric and acrocentric female only	4 (0.4)
Non-acrocentric male only	24 (2.6)
Acrocentric male only	139 (15.0)
Both non-acrocentric and acrocentric male only	6 (0.6)
Yqh + /− in males	29 (3.1)
Couples with non-acrocentric and acrocentric	143 (15.4)
Couples without polymorphism	439 (47.3)

### Reproductive outcomes according to the types of polymorphic variations

Table 3 shows details of pregnancy, miscarriage and live birth rates according to the presence or absence of chromosomal polymorphic variations. The total number of participants with chromosomal polymorphic variation was 490 (52.7%), while the remaining 439 (47.3%) did not exhibit any of the polymorphic variations.

**Table 3:** Percentages of pregnancy, miscarriage and livebirth rates of the non-acrocentric, acrocentric, Yqh in males and combination of chromosomal polymorphic variations.

Chromosomal polymorphic variations	Pregnancy rate (%)	Miscarriage rate (%)	Live birth rate (%)
Females, males or couples with polymorphic variation (n = 490)	152 (31.0)	73 (15.0)	79 (16.1)
Non-Acrocentric female only (n = 24)	7(29.2)	4 (16.7)	3 (12.5)
Acrocentric female only(n = 121)	28 (23.1)	15 (12.4)	13 (10.7)
Both non-acrocentric & acrocentric female only (n = 4)	0 (00.0)	0 (00.0)	0 (0.00)
Non-Acrocentric male only (n = 24)	8 (33.3)	2 (8.3)	6 (25.0)
Acrocentric male only (n = 139)	49 (35.2)	22 (15.8)	27 (19.4)
Both non-acrocentric & acrocentric male only (n = 6)	1 (16.7)	1 (16.7)	0 (0.00)
Yqh in males (n = 29)	8 (27.6)	3 (10.3)	5 (17.2)
Couples with non-acrocentric and acrocentric (n = 143)	51 (35.7)	26 (18.2)	25 (17.5)
Couples without polymorphic variations (n = 439)	129 (29.4)	57 (13.0)	72 (16.4)
Total (n = 929)	281 (30.2)	130 (14.0)	151 (16.2)

There were 281 pregnancies (overall pregnancy rate 30.2%; non-acrocentric 31.2%, [15/48]; acrocentric 29.6% [77/260]; combination of non-acrocentric and acrocentric 10% [1/10]; Yqh in male 27.6% [8/29]; couples with non-acrocentric and acrocentric 35.7% [51/143]; couples without polymorphism 29.4% [129/439]) recorded in 929 cycles in the study of which 130 suffered a miscarriage (overall miscarriage rate 14%; non-acrocentric 12.5%, [6/48]; acrocentric 14.2% [37/260]; combination of non-acrocentric and acrocentric 10% [1/10]; Yqh in male 10.3% [3/29]; couples with non-acrocentric and acrocentric 18.2% [26/143]; couples without polymorphism 13% [57/439] ) and 151 had a live birth (overall live birth rate 16.2%; non-acrocentric 18.7%, [9/48]; acrocentric 15.4% [40/260]; no live births in the combination of non-acrocentric and acrocentric [0/10]; Yqh in male 17.2% [5/29]; couples with non-acrocentric and acrocentric 17.5% [25/143]; couples without polymorphism 16.4% [72/439]). The total number of participants with chromosomal polymorphic variants was 490 (52.7%), while the remaining 439 (47.3%) did not exhibit any of the polymorphic variants (Table 3).

### Crude and adjusted odds ratios according to types of chromosomal polymorphic variations

Table 4 presents the crude odds ratios analysis for pregnancy, miscarriage and live birth rates according to the presence of non-acrocentric and acrocentric chromosomal polymorphic variations. The results show no evidence of an association between non-acrocentric and acrocentric chromosomal polymorphic variations and the rates of pregnancy, miscarriage, or live birth. However, confidence intervals tended to be wide allowing for substantial possibility of an association of clinically important size.

**Table 4:** Crude and adjusted odds ratio for pregnancy, miscarriage and live birth.

Outcome	Crude OR		Adjusted OR	
	Odds ratio (95% CI)	*P*	Odds ratio(95% CI)	*P*
Pregnancy				
Females, males or couples with polymorphic variations	1.08 (0.81–1.43)	0.58	1.09 (0.82–1.46)	0.52
Non-acrocentric female only	0.98 (0.40–2.44)	0.98	0.83 (0.32–2.11)	0.70
Acrocentric female only	0.72 (0.45–1.15)	0.17	0.74 (0.46–1.21)	0.23
Non-acrocentric male only	1.20 (0.50–2.87)	0.68	1.34 (0.55–3.26)	0.51
Acrocentric male only	1.30 (0.87–1.95)	0.19	1.29 (0.85–1.96)	0.22
Both acrocentric & non-acrocentric male only	0.48 (0.05–4.15)	0.50	0.45 (0.04–4.07)	0.47
Yqh in males	0.91 (0.39–2.12)	0.83	1.08 (0.46–2.56)	0.84
Couples with non-acrocentric or acrocentric	1.33 (0.89–1.98)	0.15	1.36 (0.90–2.06)	0.13
Miscarriage				
Females, males or couples with polymorphic variations	1.17 (0.80–1.70)	0.40	1.19 (0.81–1.73)	0.36
Non-acrocentric female only	1.34 (0.44–4.06)	0.60	1.25 (0.40–3.84)	0.69
Acrocentric female only	0.94 (0.51–1.74)	0.86	0.97 (0.52–1.79)	0.93
Non-acrocentric male only	0.60 (0.13–2.66)	0.51	0.64 (0.14–2.81)	0.55
Acrocentric male only	1.26 (0.73–2.14)	0.39	1.26 (0.73–2.17)	0.39
Both acrocentric & non-acrocentric male only	1.34 (0.15–11.6)	0.79	1.26 (0.14–11.1)	0.83
Yqh in males	0.77 (0.22–2.63)	0.68	0.82 (0.23–2.82)	0.75
Couples with non-acrocentric or acrocentric	1.48 (0.89–2.47)	0.12	1.51 (0.90–2.53)	0.11
Live Birth				
Females, males or couples with polymorphic variations	0.97 (0.69–1.38)	0.90	0.99 (0.69–1.42)	0.99
Non-acrocentric female only	0.72 (0.21–2.50)	0.61	0.60 (0.17–2.13)	0.43
Acrocentric female only	0.61 (0.32–1.15)	0.12	0.64 (0.33–1.21)	0.17
Non-acrocentric male only	1.69 (0.65–4.42)	0.27	2.00 (0.75–5.32)	0.16
Acrocentric male only	1.22 (0.75–2.00)	0.41	1.21 (0.73–2.00)	0.45
Yqh in males	1.06 (0.39–2.87)	0.90	1.36 (0.49–3.76)	0.55
Couples with non-acrocentric or acrocentric	1.07 (0.65–1.78)	0.76	1.08 (0.65–1.81)	0.74

The reference category is no chromosomal polymorphic variations in either partner.

There were no pregnancies, miscarriages or live births recorded in both non-acrocentric and acrocentric female only groups.

There were no live births recorded in both non-acrocentric and acrocentric male only group.

Reproductive outcomes are adjusted for confounding variables including age, BMI, serum FSH, LH levels, and type of treatment.

*OR* Odds ratio.

*CI* 95% Confidence interval.

### Number of polymorphic variations per couple

Analysis of the number of chromosomal polymorphic variations in the 929 fresh and FET cycles showed that either the female or male were carriers of one chromosomal polymorphic variation in 279 cycles (30.0%), two variations in 122 cycles (13.1%), three chromosomal polymorphic variations in 70 cycles (7.5%), four variations in 12 cycles (1.3%), and five variations in 7 cycles (0.8%). None of the partners carried a chromosomal polymorphic variation in 439 cycles (47.3%) (Table 5).

**Table 5:** Chromosomal polymorphic variations by number of variations per couple.

Categories	n (%)
One chromosomal polymorphic variation	279 (30.0)
Two chromosomal polymorphic variations	122 (13.1)
Three chromosomal polymorphic variations	70 (7.5)
Four chromosomal polymorphic variations	12 (1.3)
Five chromosomal polymorphic variations	7 (0.8)
Couples without polymorphic variations	439 (47.3)

### Reproductive outcomes according to the number of polymorphic variations

Table 6 shows the pregnancy, miscarriage and live birth rates according to number of (n = 490) either non-acrocentric or acrocentric polymorphic variations in study participants. There were 281 pregnancies (overall pregnancy rate 30.2%; one chromosomal polymorphic variation 28.7%, [80/279]; two chromosomal polymorphic variations 32.0% [39/122]; three chromosomal polymorphic variations 34.3% [24/70]; four chromosomal polymorphic variations 50.0% [6/12]; five chromosomal polymorphic variations 42.9% [3/7]; couples without polymorphism 29.4% [129/439]) recorded in 929 cycles in the study of which 130 suffered a miscarriage (overall miscarriage rate 14%; one chromosomal polymorphic variation 13.6%, [38/279]; two chromosomal polymorphic variations 13.9% [17/122]; three chromosomal polymorphic variations 21.4% [15/70]; four chromosomal polymorphic variations 16.7% [2/12]; five chromosomal polymorphic variations 14.3% [1/7]; couples without polymorphism 13% [57/439]) and 151 had a live birth (overall live birth rate 16.2%; one chromosomal polymorphic variation 15.0%, [42/279]; two chromosomal polymorphic variations 18.0% [22/122]; three chromosomal polymorphic variations 12.9% [9/70]; four chromosomal polymorphic variations 33.3% [4/12]; five chromosomal polymorphic variations 28.6% [2/7]; couples without polymorphism 16.4% [72/439]).

**Table 6:** Pregnancy, miscarriage and livebirth rates of the number of polymorphic variations in the study population.

Chromosomal polymorphic variations	n	Pregnancy rate n (%)	Miscarriage rate n (%)	Live birth rate n (%)
Number of polymorphic variations of female, male and couples (n = 490)				
One chromosomal polymorphic variation	279	80 (28.7)	38 (13.6)	42 (15.0)
Two chromosomal polymorphic variations	122	39 (32.0)	17 (13.9)	22 (18.0)
Three chromosomal polymorphic variations	70	24 (34.3)	15 (21.4)	9 (12.9)
Four chromosomal polymorphic variations	12	6 (50.0)	2 (16.7)	4 (33.3)
Five chromosomal polymorphic variations	7	3 (42.9)	1 (14.3)	2 (28.6)
Non-carriers of polymorphic variations	439	129 (29.4)	57 (13.0)	72 (16.4)
Total	929	281 (30.2)	130 (14.0)	151 (16.2)

### Crude and adjusted odds ratios according to the number of polymorphic variations

Table 7 shows the crude and adjusted odds ratios for pregnancy, miscarriage and live birth rates according to number of polymorphic variations. We found no evidence of an association between number of chromosomal polymorphic variations and these reproductive outcomes. But again, confidence intervals are wide.

**Table 7:** Crude and adjusted odds ratio for pregnancy of the number of polymorphic variations in the study population.

Outcome	Crude OR		Adjusted OR	
	Odds ratio (95% CI)	*P*	Odds ratio (95% CI)	*P*
Pregnancy				
One chromosomal polymorphic variation	0.96 (0.69–1.34)	0.83	0.96 (0.68–1.35)	0.85
Two chromosomal polymorphic variations	1.12 (0.73–1.73)	0.58	1.18 (0.75–1.83)	0.46
Three chromosomal polymorphic variations	1.25 (0.73–2.13)	0.40	1.25 (0.72–2.17)	0.42
Four chromosomal polymorphic variations	2.40 (0.76–7.58)	0.13	2.49 (0.75–8.20)	0.13
Five chromosomal polymorphic variations	1.80 (0.39–8.16)	0.44	2.20 (0.47–10.33)	0.31
Miscarriage				
One chromosomal polymorphic variation	1.05 (0.67–1.64)	0.80	1.05 (0.67–1.65)	0.79
Two chromosomal polymorphic variations	1.08 (0.60–1.94)	0.78	1.12 (0.62–2.01)	0.70
Three chromosomal polymorphic variations	1.82 (0.96–3.44)	0.06	1.89 (0.99–3.60)	0.05
Four chromosomal polymorphic variations	1.34 (0.28–6.27)	0.71	1.34 (0.28–6.40)	0.71
Five chromosomal polymorphic variations	1.11 (0.13–9.44)	0.91	1.10 (0.12–9.47)	0.92
Livebirth				
One chromosomal polymorphic variation	0.90 (0.59–1.36)	0.63	0.91 (0.60–1.40)	0.69
Two chromosomal polymorphic variations	1.12 (0.66–1.89)	0.67	1.17 (0.68–2.01)	0.55
Three chromosomal polymorphic variations	0.75 (0.35–1.58)	0.45	0.71 (0.33–1.52)	0.38
Four chromosomal polymorphic variations	2.54 (0.74–8.68)	0.13	2.57 (0.72–9.14)	0.14
Five chromosomal polymorphic variations	2.03 (0.38–10.71)	0.40	2.93 (0.53–16.12)	0.21

The reference category is no chromosomal polymorphic variations in either partner.

Reproductive outcomes are adjusted for confounding variables including age, BMI, serum FSH, LH levels, and type of treatment.

*OR* Odds ratio.

*CI* 95% Confidence intervals.

## Discussion

We analysed data from 929 fresh and frozen embryo transfer cycles in 692 women who underwent karyotyping analysis prior to the ICSI procedure. We found no evidence of a difference in pregnancy, miscarriage, or live birth rates between participants with any type or number of chromosomal polymorphic variation (female with non-acrocentric, acrocentric and their combinations, male with non-acrocentric, acrocentric and their combination, Yqh in males, couples with non-acrocentric and acrocentric polymorphic variations) and those with no chromosomal polymorphic variations. However, the confidence intervals were often wide and allow for a substantial possibility of an association of clinically important size.

Our study is unique in that we found no evidence of an association in the study population, and any effect on reproductive outcomes is likely to be minimal. We followed up all recruited participants up to live birth or and adjusted results for a number of potential confounders. However, there was a low prevalence of chromosomal polymorphic variations in this study, which may have led to insufficient power to detect clinically meaningful effects. Therefore, the sample size does not sufficient to draw a conclusion representing the total Sri Lankan population.

Embryos’ ability to self-correct genetic abnormalities may explain why chromosomal polymorphic variations do not seem to result in adverse reproductive outcomes. A recent study shed insight into human embryogenesis and the ability of self-correction, suggesting that genetic abnormalities may resolve during the initial stages of cell divisions up to implantation^20^. It is reasonable to postulate that through increased cell proliferation and cell death, mosaic embryos may be more prone to self-correction in comparison to both euploidy and aneuploidy embryos^21,22^. Animal studies have demonstrated that embryos during the preimplantation development to overcome chromosomal instability, encapsulate chromosome containing fragments in to micronuclei and their elimination through cellular fragmentation^23,24^. The balance between cell survival and apoptosis is controlled by a cell death programme that eliminates damaged cells in early embryo development^25^. Therefore, through self-correction, chromosomal polymorphic variations may lead to similar treatment outcomes in carrier couples.

There is evidence suggesting that ICSI may lead to a better chance of a clinical pregnancy compared to conventional IVF in the presence of acrocentric chromosomal polymorphisms. The Intra cytoplasmic sperm injection has increased compared to standard IVF, with double the number of cycles globally. ICSI has an advantage of selecting the progressive, morphologically normal spermatozoa and higher fertilization rates than standard IVF procedures^26^. However, it is not clear how ICSI could lead to better chance of a clinical pregnancy compared to conventional IVF in the presence of acrocentric chromosomal polymorphism^17^. Future research could investigate whether there is an advantage in ICSI treatment compared to standard in vitro fertilization in the presence of chromosomal polymorphic variations.

Our data, in contrast to previous reports, do not support a deleterious effect of the type or number of chromosomal polymorphic variations in females, males or couples on their reproductive outcomes. We found no association in the Sri Lankan population, and any association with reproductive outcomes is likely to be non-influential in the reproductive outcome. The karyotyping analysis as a standard method will provide valuable information of the parental cytogenetic abnormalities which will contribute to genetic counselling prior to IVF or ICSI procedure. Although, the chromosomal polymorphic variations do not influence the reproductive outcome of ICSI, routine karyotyping could detect other abnormalities such as chromosomal aberrations. Therefore, it facilitates counselling of the couples and to offer pre-implantation genetic testing with structural chromosomal rearrangements (PGT-SR) to avoid passing the chromosomal abnormality to the offspring. Hence, routine karyotyping may still have a role outside the research context.

In addition, additional prospective, adequately powered studies, conducted in multiethnic populations, are required to further investigate whether the detection of chromosomal polymorphic variants prior to assisted conception may in fact be a futile diagnostic tool.

## Supplementary Information

Supplementary Information.

## Data Availability

The datasets generated during and/or analyzed during the current study are available in the Harvard Dataverse repository, https://doi.org/10.7910/DVN/51HZOP.
