# Author Correction: A comprehensive analysis of chromosomal polymorphic variants on reproductive outcomes after intracytoplasmic sperm injection treatment

**DOI:** 10.1038/s41598-023-36732-x

**Published:** 2023-06-21

**Authors:** Madara S. B. Ralapanawe, Sugandika L. Gajaweera, Nishendra Karunaratne, Vajira H. W. Dissanayake, Malcolm J. Price, Pedro Melo, Arri Coomarasamy, Ioannis D. Gallos

**Affiliations:** 1 Tommy’s National Centre for Miscarriage Research, Institute of Metabolism and Systems Research, Institute of Translational Medicine (ITM), University of Birmingham, 4th Floor, Edgbaston, Birmingham, B15 2TT UK; 2 Fertility Centre, Lanka Hospitals Corporation Plc, 578, Elvitigala Mawatha, Colombo, 00500 Sri Lanka; 3 Department of Anatomy, Genetics and Biomedical Informatics, Faculty of Medicine, University of Colombo, Colombo, 00800 Sri Lanka; 4 Institute of Applied Health Research, University of Birmingham, Birmingham, B15 2TT UK; 5 NIHR Birmingham Biomedical Research Centre, University Hospitals Birmingham NHS Foundation Trust, University of Birmingham, Birmingham, B15 2TH UK

Correction to: *Scientific Reports* 10.1038/s41598-023-28552-w, published online 24 January 2023

The original version of this Article contained errors in the spelling of the author Vajira H.W. Dissanayake, which was incorrectly given as Vajira H.W. Dissnayake.

The original Article has been corrected.

