# Supplementary material for: A comprehensive analysis of chromosomal polymorphic variants on reproductive outcomes after intracytoplasmic sperm injection treatment
Source: Sci Rep. 2023 Jan 24;13:1319. doi: 10.1038/s41598-023-28552-w (PMC9873903; doi:10.1038/s41598-023-28552-w)
Supplement: Supplementary file 1 — Supplementary Information. [file 41598_2023_28552_MOESM1_ESM.docx]

**Supplementary Table 1** Distribution of chromosomal polymorphic variants in female and male partners in the study population

| Classification | Female karyotype | n | Male karyotype | n |
| --- | --- | --- | --- | --- |
| Chromosomal polymorphic variants in female partners only (n=149) | | | | |
| Non-acrocentric Polymorphic variants | | | | |
| qh+ | 1qh+ | 1 | 46XY | 1 |
|  | 9qh+ | 20 | 46XY | 20 |
|  | 9qh- | 2 | 46XY | 2 |
|  | 16qh+ | 1 | 46XY | 1 |
| Acrocentric Polymorphic variants | | | | |
| One acrocentric polymorphic variant | | | | |
| pstk+, ps+, cenh+ | 13pstk+ | 11 | 46XY | 11 |
|  | 14pstk+ | 15 | 46XY | 15 |
|  | 15pstk+ | 28 | 46XY | 28 |
|  | 21pstk+ | 15 | 46XY | 15 |
|  | 22pstk+ | 23 | 46XY | 23 |
|  | 13ps+ | 1 | 46XY | 1 |
|  | 15ps+ | 2 | 46XY | 2 |
|  | 22ps+ | 1 | 46XY | 1 |
|  | 15cenh+ | 1 | 46XY | 1 |
| Two acrocentric polymorphic variants | | | | |
| pstk+, ps+ | 13pstk+, 14pstk+ | 1 | 46XY | 1 |
|  | 13pstk+, 15pstk+ | 1 | 46XY | 1 |
|  | 13pstk+, 21pstk+ | 4 | 46XY | 4 |
|  | 13pstk+, 22pstk+ | 2 | 46XY | 2 |
|  | 14pstk+, 15pstk+ | 1 | 46XY | 1 |
|  | 14pstk+, 21pstk+ | 2 | 46XY | 2 |
|  | 14pstk+, 22pstk+ | 2 | 46XY | 2 |
|  | 15pstk+, pstk+ | 4 | 46XY | 4 |
|  | 15pstk+, 22pstk+ | 4 | 46XY | 4 |
|  | 21pstk+, 22pstk+ | 2 | 46XY | 2 |
|  | 13ps+, 22pstk+ | 1 | 46XY | 1 |
| Combination of non-acrocentric and acrocentric polymorphic variants | | | | |
| qh+, pstk+, ps+ | 9qh+, 13pstk+ | 1 | 46XY | 1 |
|  | 9qh+, 21pstk+ | 2 | 46XY | 2 |
|  | 9qh+, 14ps+, 21 ps+, 22ps+ | 1 | 46XY | 1 |
|  |  |  |  |  |
| Chromosomal Polymorphic variants in male partners only (n=198) | | | | |
| Non-acrocentric Polymorphic variants | | | | |
| qh+/qh- | 46XX | 2 | 1qh+ | 2 |
|  | 46XX | 1 | 1qh- | 1 |
|  | 46XX | 17 | 9qh+ | 17 |
| Inv(9) | 46XX | 4 | Inv(9) (p12q13) | 4 |
| Acrocentric Polymorphic variants | | | | |
| One acrocentric polymorphic variant | | | | |
| pstk+, ps+, cenh+ | 46XX | 12 | 13pstk+ | 12 |
|  | 46XX | 13 | 14pstk+ | 13 |
|  | 46XX | 26 | 15pstk+ | 26 |
|  | 46XX | 29 | 21pstk+ | 29 |
|  | 46XX | 19 | 22pstk+ | 19 |
|  | 46XX | 2 | ps+ | 2 |
|  | 46XX | 4 | 13ps+ | 4 |
|  | 46XX | 2 | 14ps+ | 2 |
|  | 46XX | 1 | 15ps+ | 1 |
|  | 46XX | 3 | 21ps+ | 3 |
|  | 46XX | 6 | 22ps+ | 6 |
|  | 46XX | 1 | 15cenh+ | 1 |
| Two acrocentric polymorphic variants | | | | |
| pstk+, ps+ | 46XX | 2 | 13pstk+, 14pstk+ | 2 |
|  | 46XX | 1 | 13pstk+, 21pstk+ | 1 |
|  | 46XX | 3 | 13pstk+, 22pstk+ | 3 |
|  | 46XX | 4 | 14pstk+, 21pstk+ | 4 |
|  | 46XX | 2 | 15pstk+, 21pstk+ | 2 |
|  | 46XX | 1 | 15ps+, 22pstk+ | 1 |
|  | 46XX | 1 | 14pstk+,21ps+ | 1 |
|  | 46XX | 3 | 21pstk+, 22pstk+ | 3 |
|  | 46XX | 1 | 22pstk+, pstk+ | 1 |
| Three acrocentric polymorphic variants | | | | |
| pstk+, ps+ | 46XX | 1 | 13pstk+, 14pstk+, 21pstk+ | 1 |
|  | 46XX | 1 | 13pstk+, 15pstk+, 21pstk+ | 1 |
|  | 46XX | 1 | 14pstk+, 14pstk+, 22pstk+ | 1 |
| Combination of non-acrocentric and acrocentric polymorphic variants | | | | |
| qh+/-, pstk+ | 46XX | 1 | 9qh+, 15pstk+ | 1 |
|  | 46XX | 1 | 9qh+, 21pstk+ | 1 |
|  | 46XX | 2 | 9qh-, 14pstk+ | 2 |
|  | 46XX | 2 | 9qh-, 21pstk+ | 2 |
| Male Yqh | | | | |
| Male Yqh only | | | | |
| Yqh+/- | 46XX | 13 | Yqh+ | 13 |
|  | 46XX | 5 | Yqh- | 5 |
| Male Yqh and one acrocentric polymorphic variant | | | | |
| Yqh+/-, pstk+ | 46XX | 1 | Yqh+, 15pstk+ | 1 |
|  | 46XX | 2 | Yqh-, 14pstk+ | 2 |
|  | 46XX | 2 | Yqh-, 21pstk+ | 2 |
|  | 46XX | 2 | Yqh-, 22pstk+ | 2 |
| Male Yqh and two acrocentric polymorphic variants | | | | |
| Yqh+/-, pstk+ | 46XX | 3 | Yqh+, 13pstk+, 15pstk+ | 3 |
|  | 46XX | 1 | Yqh-, 13pstk+, 15pstk+ | 1 |
| Couples with chromosomal polymorphic variants (n=143) | | | | |
| Non-acrocentric polymorphic variant in female and acrocentric polymorphic variants in male | | | | |
| qh+/-, pstk+, ps+ | 1qh+ | 1 | 13pstk+ | 1 |
|  | 9qh+ | 2 | 13pstk+ | 2 |
|  | 9qh+ | 1 | 14pstk+ | 1 |
|  | 9qh+ | 2 | 15pstk+ | 2 |
|  | 9qh+ | 1 | 22pstk+ | 1 |
|  | 9qh- | 2 | 22pstk+ | 2 |
|  | 9qh+ | 2 | 13pstk+, 14pstk+ | 2 |
|  | 9qh+ | 2 | 21ps+ | 2 |
|  | 16qh+ | 1 | 14pstk+, 21pstk+ | 1 |
| Non-acrocentric polymorphic variants in both female and male | | | | |
| qh+/- | 9qh+ | 1 | 9qh+ | 1 |
| Non-acrocentric polymorphic variants in male and acrocentric polymorphic variants in female | | | | |
| qh+/-, inv (9), pstk+, ps+ | 14pstk+ | 1 | 9qh- | 1 |
|  | 21pstk+ | 1 | 16qh+ | 1 |
|  | 14pstk+, 15pstk+ | 1 | 9qh+ | 1 |
|  | 13pstk+, 15pstk+, 22pstk+ | 2 | 9qh+ | 2 |
| Combination of non-acrocentric and acrocentric polymorphic variants in female and male | | | | |
| qh+/-, inv(9), pstk+, ps+ | 13pstk+ | 2 | 9qh-, 21pstk+ | 2 |
|  | 14pstk+ | 1 | 9qh-, 13pstk+, 21pstk+ | 1 |
|  | 15pstk+ | 1 | 9(inv), 13pstk+ | 1 |
|  | 21pstk+ | 3 | 9qh+, 13pstk+ | 3 |
|  | 22pstk+ | 2 | 9qh+, 13pstk+ | 2 |
|  | 22pstk+ | 1 | 9qh-, 14pstk+ | 1 |
|  | 22pstk+ | 1 | 9qh+, 21pstk+ | 1 |
|  | 14pstk+, 15pstk+ | 1 | 9qh+, 13pstk+ | 1 |
|  | 13pstk+, 15pstk+ | 1 | 9qh-, 13pstk+, 14ps+ | 1 |
|  | 15pstk+, ps+ | 1 | 9qh+, 13pstk+, 22pstk+ | 1 |
|  | 9qh-, 14pstk+ | 2 | 9qh+, 13pstk+, 21pstk+ | 2 |
|  | 9qh+, 15pstk+ | 1 | 14pstk+ | 1 |
|  | 9qh+, 22pstk+ | 1 | 9qh+ | 1 |
| One acrocentric polymorphic variant in female and male | | | | |
| pstk+, ps+ | 13pstk+ | 1 | 13pstk+ | 1 |
|  | 13pstk+ | 1 | 14pstk+ | 1 |
|  | 13pstk+ | 2 | 15pstk+ | 2 |
|  | 13pstk+ | 4 | 21pstk+ | 4 |
|  | 13pstk+ | 2 | 22pstk+ | 2 |
|  | 14pstk+ | 1 | 13pstk+ | 1 |
|  | 14pstk+ | 1 | 14pstk+ | 1 |
|  | 14pstk+ | 3 | 15pstk+ | 3 |
|  | 14pstk+ | 1 | 21pstk+ | 1 |
|  | 15pstk+ | 1 | 14pstk+ | 1 |
|  | 15pstk+ | 1 | 22pstk+ | 1 |
|  | 21pstk+ | 1 | 14pstk+ | 1 |
|  | 21pstk+ | 1 | 15pstk+ | 1 |
|  | 21pstk+ | 3 | 21pstk+ | 3 |
|  | 21pstk+ | 1 | 22pstk+ | 1 |
|  | 22pstk+ | 2 | 13pstk+ | 2 |
|  | 22pstk+ | 3 | 15pstk+ | 3 |
|  | 21pstk+ | 1 | 13ps+ | 1 |
|  | 22pstk+ | 1 | 13ps+ | 1 |
|  | 14ps+ | 2 | 14pstk+ | 2 |
|  | 14ps+ | 2 | 15pstk+ | 2 |
|  | 14ps+ | 1 | 14ps+ | 1 |
| One acrocentric polymorphic variant in female and two acrocentric polymorphic variants in male | | | | |
| pstk+, ps+ | 13pstk+ | 1 | 13pstk+, 21pstk+ | 1 |
|  | 13pstk+ | 2 | 14pstk+, 21pstk+ | 2 |
|  | 13pstk+ | 1 | 21pstk+, 22pstk+ | 1 |
|  | 14pstk+ | 3 | 13pstk+, 22pstk+ | 3 |
|  | 14pstk+ | 2 | 14pstk+, 22pstk+ | 2 |
|  | 14pstk+ | 1 | 13pstk+, 22ps+, pstk+ | 1 |
|  | 15pstk+ | 1 | 15pstk+, 21pstk+ | 1 |
|  | 15pstk+ | 2 | 14pstk+, 22pstk+ | 2 |
|  | 15pstk+ | 2 | 22pstk+, ps+ | 2 |
|  | 21pstk+ | 2 | 13pstk+, 15pstk+ | 2 |
|  | 21pstk+ | 1 | 14pstk+, 15pstk+ | 1 |
|  | 21pstk+ | 1 | 22pstk+, ps+ | 1 |
|  | 22pstk+ | 1 | 14pstk+, 15pstk+ | 1 |
|  | 22pstk+ | 2 | 15pstk+, 21pstk+ | 2 |
| Two acrocentric polymorphic variants in female and one acrocentric polymorphic variant in male | | | | |
| pstk+, ps+ | 13pstk+, 15pstk+ | 1 | 22pstk+ | 1 |
|  | 13pstk+, 21pstk+ | 1 | 14pstk+ | 1 |
|  | 13pstk+, 21pstk+ | 2 | 21pstk+ | 2 |
|  | 13pstk+, 22pstk+ | 1 | 14pstk+ | 1 |
|  | 13pstk+, 22pstk+ | 2 | 21pstk+ | 2 |
|  | 13pstk+, 15pstk+ | 1 | 22ps+ | 1 |
|  | 14pstk+, 15pstk+ | 1 | 22pstk+ | 1 |
|  | 14pstk+, 21pstk+ | 1 | 13pstk+ | 1 |
|  | 14pstk+, 21pstk+ | 3 | 15pstk+ | 3 |
|  | 14pstk+, 21pstk+ | 2 | 21pstk+ | 2 |
|  | 15pstk+, 22pstk+ | 4 | 14pstk+ | 4 |
| Two acrocentric polymorphic variants in female and in male | | | | |
| pstk+ | 14pstk+, 21pstk+ | 1 | 15pstk+, 22pstk+ | 1 |
|  | 14pstk+, 21pstk+ | 1 | 14pstk+, 22pstk+ | 1 |
|  | 15pstk+, 21pstk+ | 1 | 14pstk+, 21pstk+ | 1 |
|  | 21pstk+, 22pstk+ | 1 | 13pstk+, 14pstk+ | 1 |
| Three acrocentric polymorphic variants in female and one/two acrocentric polymorphic variants in male | | | | |
| pstk+, ps+ | 14pstk+, ps+, 22pstk+ | 1 | 22pstk+, ps+ | 1 |
|  | 14pstk+, 15pstk+, 21pstk+ | 1 | 14ps+, pstk+ | 1 |
|  | 14pstk+, 15pstk+, 22pstk+ | 1 | 15pstk+ | 1 |
|  | 14pstk+, 15pstk+, 22pstk+ | 1 | 13pstk+, 21pstk+ | 1 |
| Non-acrocentric polymorphic variant in female and Male Yqh | | | | |
| qh+/-, Yqh+/- | 9qh- | 1 | Yqh- | 1 |
| Acrocentric polymorphic variant in female and Male Yqh | | | | |
| pstk+, ps+, Yqh+/- | 13pstk+ | 1 | Yqh+ | 1 |
|  | 14pstk+ | 1 | Yqh- | 1 |
|  | 15pstk+ | 2 | Yqh+ | 2 |
|  | 21pstk+ | 2 | Yqh+ | 2 |
|  | 21pstk+ | 2 | Yqh- | 2 |
|  | 14ps+ | 2 | Yqh- | 2 |
|  | 22ps+ | 2 | Yqh+ | 2 |
| Combination of non-acrocentric and acrocentric polymorphic variants in female, male and male Yqh | | | | |
| pstk+, Yqh+/- | 14pstk+ | 1 | Yqh-, 13pstk+ | 1 |
|  | 15pstk+ | 1 | Yqh-, 22pstk+ | 1 |
|  | 15pstk+ | 1 | Yqh+, 22pstk+ | 1 |
|  | 22pstk+ | 2 | Yqh+, 21pstk+ | 2 |
|  | 9qh+, 22pstk+ | 1 | Yqh-, 14pstk+ | 1 |
|  | 15pstk+, 21pstk+ | 1 | Yqh- | 1 |
